# Supplementary material for: Morphological and Compositional Features of Chronic Internal Carotid Artery Occlusion in MR Vessel Wall Imaging Predict Successful Endovascular Recanalization
Source: Diagnostics (Basel). 2023 Jan 1;13(1):147. doi: 10.3390/diagnostics13010147 (PMC9818158; doi:10.3390/diagnostics13010147)

Figure S1: Study flowchart

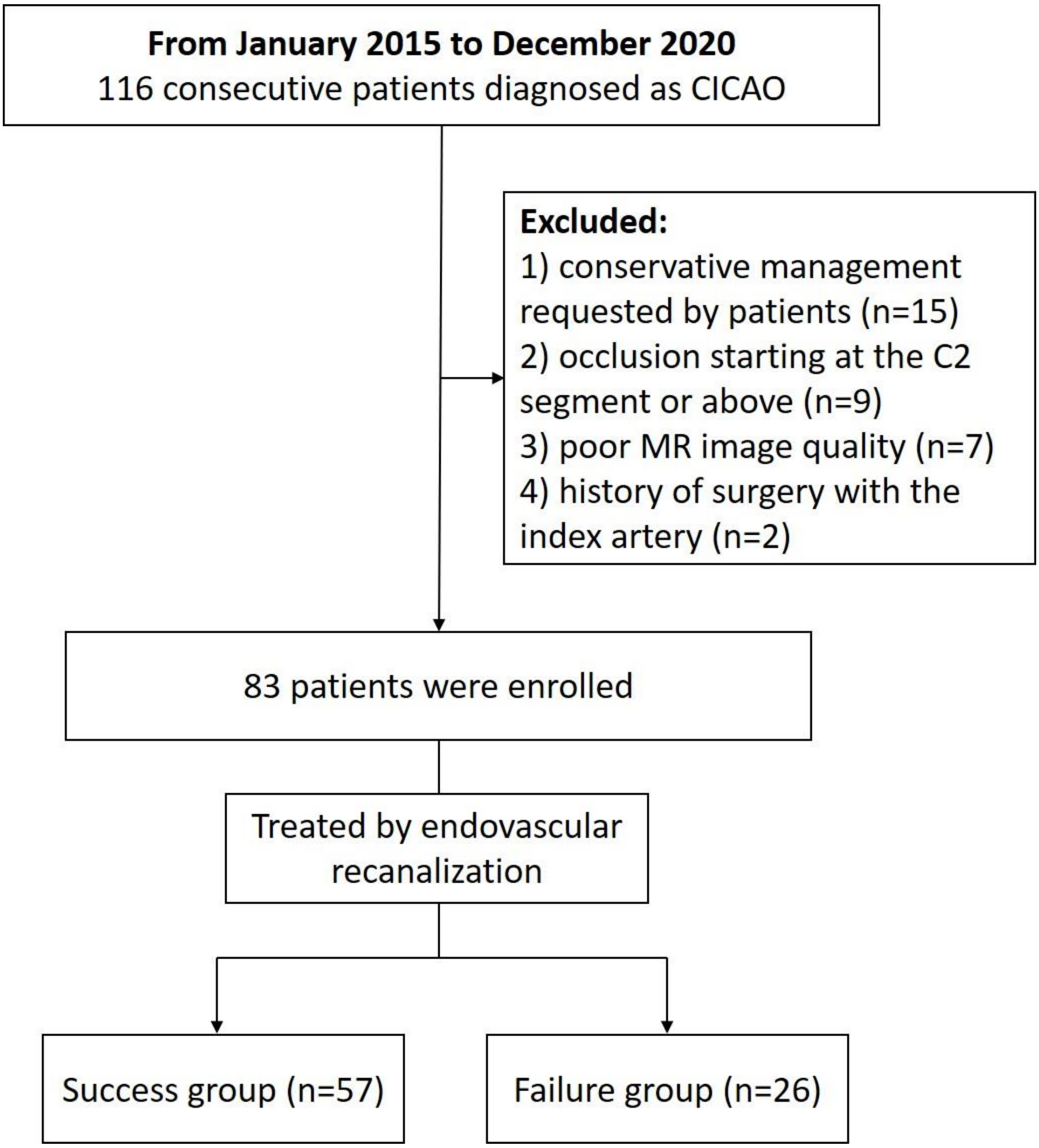

Figure S2: Success rates of endovascular recanalization according to the MR CICAO score

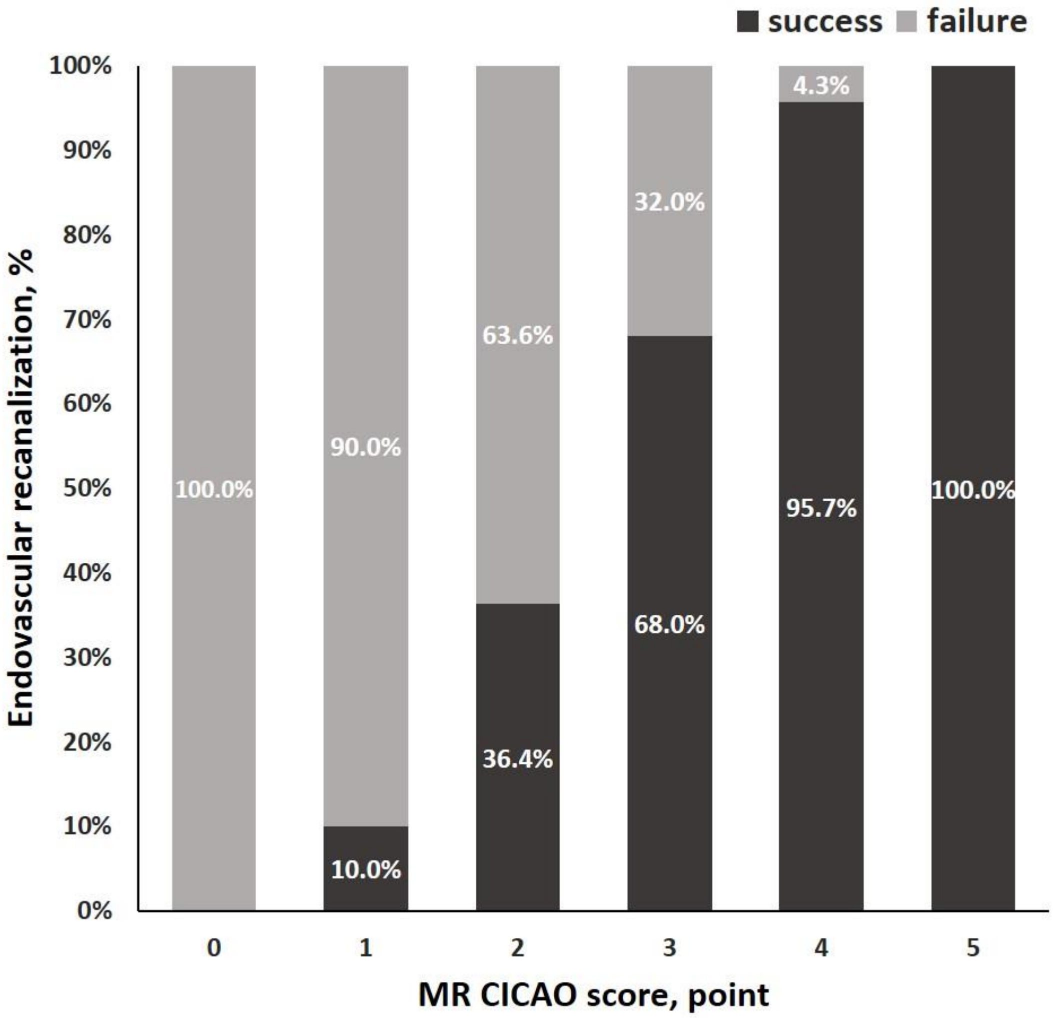

Supplement: Supplementary file 1 [file diagnostics-13-00147-s001.zip › diagnostics-2087135-supplementary materials.pdf]
